# A comparison of the ability of rilpivirine (TMC278) and selected analogues to inhibit clinically relevant HIV-1 reverse transcriptase mutants

**DOI:** 10.1186/1742-4690-9-99

**Published:** 2012-12-05

**Authors:** Barry C Johnson, Gary T Pauly, Ganesha Rai, Disha Patel, Joseph D Bauman, Heather L Baker, Kalyan Das, Joel P Schneider, David J Maloney, Eddy Arnold, Craig J Thomas, Stephen H Hughes

**Affiliations:** 1 HIV Drug Resistance Program, National Cancer Institute, Frederick National Laboratory for Cancer Research, P.O. Box B, Building 539, Room 130A, Frederick, MD, 21702-1201, USA; 2 Chemical Biology Laboratory, Frederick National Laboratory for Cancer Research, Frederick, MD, USA; 3 NIH Chemical Genomics Center, National Center for Advancing Translational Sciences, NIH, 9800 Medical Center Drive, Bethesda, MD, MSC 3370, USA; 4 Center for Advanced Biotechnology and Medicine and Department of Chemistry and Chemical Biology, Rutgers University, Piscataway, NJ, USA

**Keywords:** HIV, Reverse transcriptase, Rilpivirine

## Abstract

**Background:**

The recently approved anti-AIDS drug rilpivirine (TMC278, Edurant) is a nonnucleoside inhibitor (NNRTI) that binds to reverse transcriptase (RT) and allosterically blocks the chemical step of DNA synthesis. In contrast to earlier NNRTIs, rilpivirine retains potency against well-characterized, clinically relevant RT mutants. Many structural analogues of rilpivirine are described in the patent literature, but detailed analyses of their antiviral activities have not been published. This work addresses the ability of several of these analogues to inhibit the replication of wild-type (WT) and drug-resistant HIV-1.

**Results:**

We used a combination of structure activity relationships and X-ray crystallography to examine NNRTIs that are structurally related to rilpivirine to determine their ability to inhibit WT RT and several clinically relevant RT mutants. Several analogues showed broad activity with only modest losses of potency when challenged with drug-resistant viruses. Structural analyses (crystallography or modeling) of several analogues whose potencies were reduced by RT mutations provide insight into why these compounds were less effective.

**Conclusions:**

Subtle variations between compounds can lead to profound differences in their activities and resistance profiles. Compounds with larger substitutions replacing the pyrimidine and benzonitrile groups of rilpivirine, which reorient pocket residues, tend to lose more activity against the mutants we tested. These results provide a deeper understanding of how rilpivirine and related compounds interact with the NNRTI binding pocket and should facilitate development of novel inhibitors.

## Background

Human immunodeficiency virus type 1 (HIV-1), which causes acquired immunodeficiency syndrome (AIDS), poses important problems for world health with an estimated 2.7 million newly infected individuals in 2010 and an estimated 34 million individuals living with HIV/AIDS worldwide [1]. However, progress is being made in treating HIV/AIDS. Highly active antiretroviral therapy (HAART) is reaching a large number of infected individuals that, until recently, either had no access to treatment, or relied upon ineffective monotherapies. Modern HAART regimens consist of a combination of drugs from several classes that can include nucleoside reverse transcriptase inhibitors (NRTIs), integrase inhibitors, entry inhibitors, CCR5 antagonists, protease inhibitors and non-nucleoside reverse transcriptase inhibitors (NNRTIs) [2]. NNRTIs are used as components of HAART regimens because of their high specificity and modest toxicity [3].

**Figure 1:**
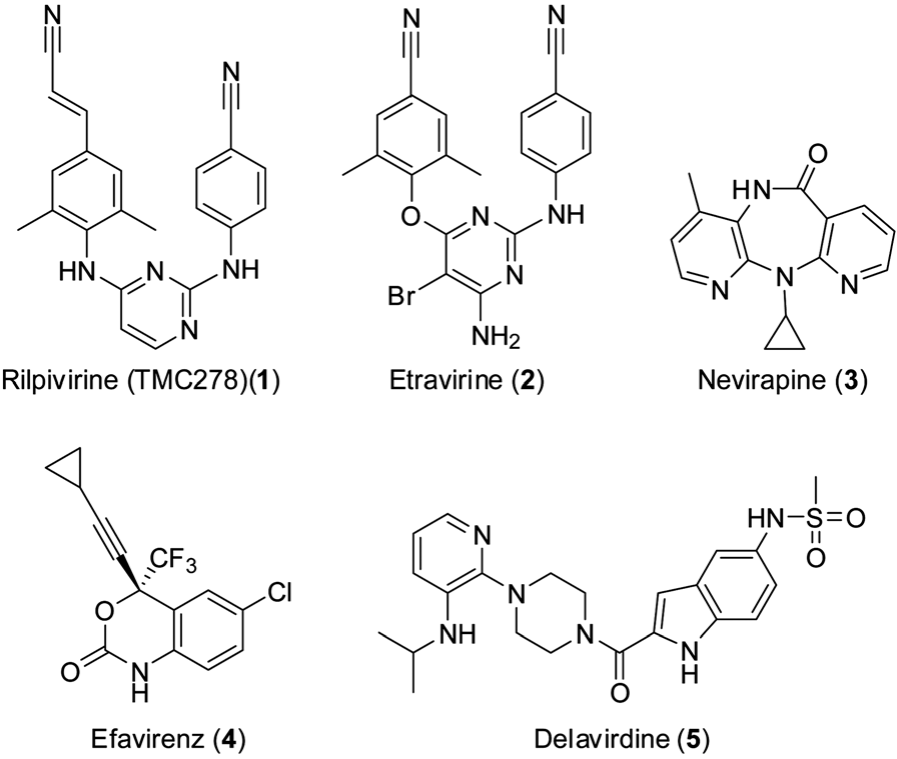
Chemical structures of approved NNRTIs rilpivirine (1), etravirine (2), nevirapine (3), efavirenz (4) and delaviridine (5).

At present, five NNRTIs have been approved for use in humans by the U.S. Food and Drug Administration: rilpivirine (TMC278) (**1**), etravirine (**2**), nevirapine (**3**), efavirenz (**4**) and delavirdine (**5**) (Figure 1). Multiple other chemotypes that have NNRTI activity have been reported, including numerous structurally unique small molecules. These compounds bind in the same hydrophobic binding pocket, and the literature shows a distinct progression in NNRTI design [4-7]. Notably, the (*E*)-3-(4-amino-3,5-dimethylphenyl)acrylonitrile ring system of rilpivirine (Figure 1, top left ring system of **1**) led to improved potency against both WT and mutant RTs.

Although HIV-1 can develop resistance to all of the approved drugs, the first generation NNRTIs were particularly susceptible to the rapid emergence of resistant virus [8]. Rilpivirine and etravirine belong to the chemical class of NNRTIs known as diarylpyrimidines (DAPYs) that was developed through the collaborative efforts of pharma and academia [3,9,10]. The DAPY class of NNRTIs was selected for further study because these compounds are able to bind and inhibit WT RT and a number of clinically relevant NNRTI-resistant mutants. This ability derives from the strategic flexibility of the compounds within the NNRTI binding pocket [11]. Further investigation of the DAPY compounds remains important because they retain activity against a wide range of drug-resistant mutants [12].

Clinical trials of rilpivirine, in combination with two NRTIs, showed that distinct patterns of mutants were selected in patients experiencing virologic failure (VF) compared to treatment regimens with efavirenz [13,14]. Patients receiving rilpivirine were more likely to harbor viruses with NRTI resistance mutations, particularly M184I, than patients receiving efavirenz. Among the NNRTI resistance mutations, E138K was selected most frequently with rilpivirine, while K103N was selected most frequently with efavirenz. The association between M184I and E138K selection in patients receiving rilpivirine is noteworthy, because E138K has been shown to restore replication capacity to viruses harboring M184 mutations [15]. E138K and M184I were detected in 45% and 47%, respectively, of patients receiving rilpivirine and experiencing VF [13]. These mutations were found in 0% and 7%, respectively, of patients experiencing VF in the efavirenz group. By comparison, clinical trials revealed that resistance to etravirine is achieved by a combination of at least three mutations, including V90I, A98G, L100I, K101E/P, V106I, V179D/F, Y181C/I/V and G190A/S [16]. There has been considerable work showing how the DAPY class of NNRTIs bind and inhibit RT [3,10,17-20]. Several groups have designed derivatives of these compounds [20,21]. However, much of the SAR surrounding this class of agents is found primarily in the patent literature.

**Figure 2:**
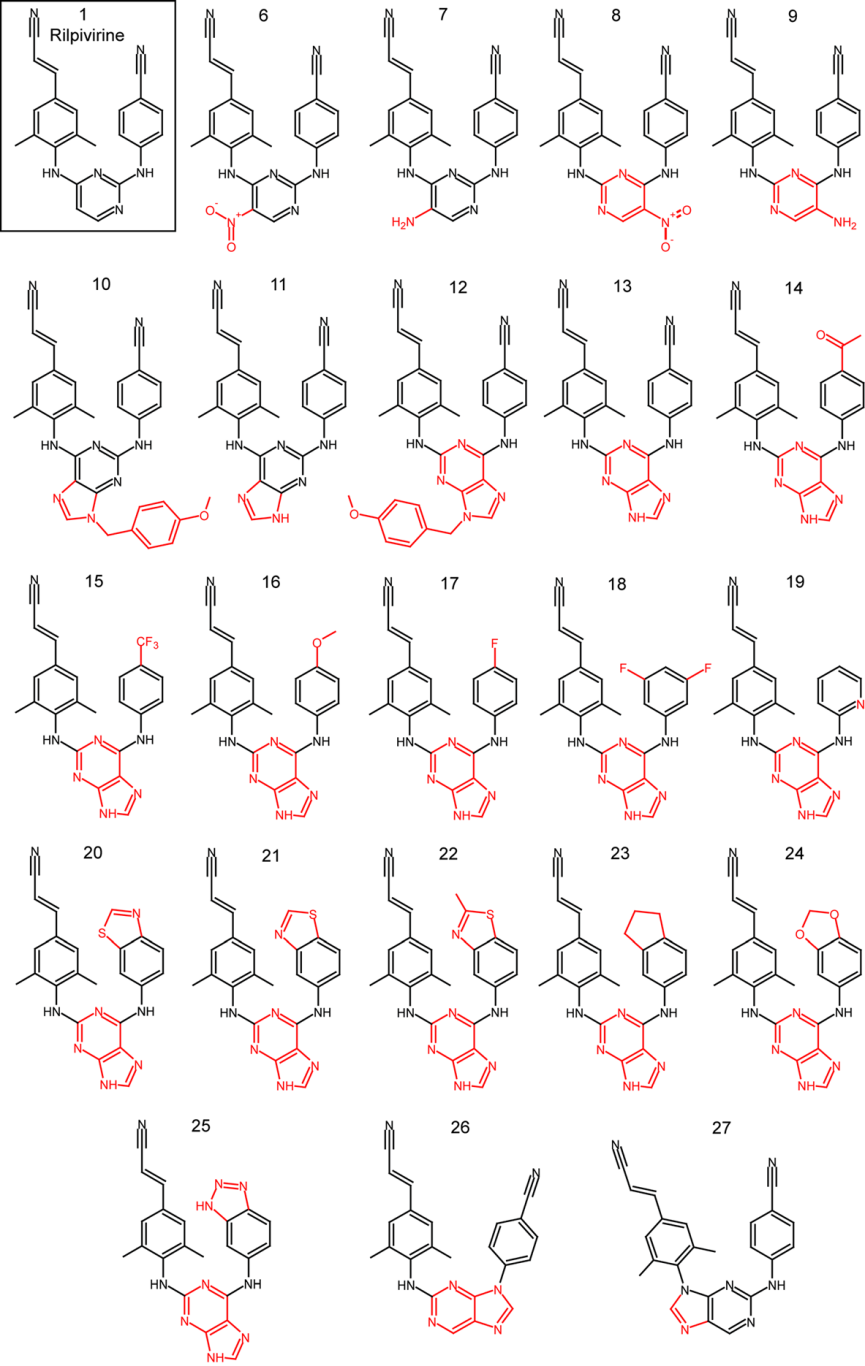
**Chemical structures of rilpivirine analogues.** Analogues differed from rilpivirine (**1**, boxed) by the addition of an exocyclic moiety to the pyrimidine ring in either of its ‘flipped’ conformations (**6**–**9**), the replacement of the pyrimidine ring with a 2,6-purine ring system in ‘flipped’ conformations with or without a protecting group (**10**–**13**), replacing the 4-benzonitrile moiety in addition to replacing the pyrimidine ring with a 2,6-purine (**14**–**25**), or replacing the pyrimidine ring with a 2,9-purine ring system (**26**, **27**). Differences between each analogue and rilpivirine are indicated in red.

The activities of a number of key structural analogues of both rilpivirine (**1**) and etravirine (**2**) against NNRTI-resistant mutants have not been reported, nor have the interactions of these analogs with HIV-1 RT been evaluated in structural studies. Such information would provide a deeper understanding of how these molecules inhibit WT and drug-resistant RTs and how they can be engineered to enhance their activity against viruses that carry resistance mutations. In addition, novel analogues that select for different patterns of mutations than rilpivirine or etravirine could be used as second-line treatment in patients experiencing VF with either of these drugs, or possibly, in combination with existing drugs. The difference in mutation profile selected by these drugs, despite their structural similarities, suggests that subtle changes can significantly affect the mechanism by which resistance arises. To pursue the goal of identifying analogues that will select novel resistance mutations, we focused our efforts on compounds with specific alterations of the pyrimidine moiety and the 4-amino-benzonitrile moiety of rilpivirine (**1**) (Figure 2). We were motivated to alter only these structural motifs because the (*E*)-3-(4-amino-3,5-dimethylphenyl)acrylonitrile ring system has been shown to be an important motif for rilpivirine binding within the induced NNRTI-binding pocket [19].

## Results and discussion

### Inhibitory activity of pyrimidine-based analogues

We examined the effects of rilpivirine and the analogues on HIV-1 replication using a previously described assay and report the concentrations at which luciferase reporter activity was reduced by half relative to uninhibited controls (EC_50_) with standard deviations in parentheses. In the assay, viral vectors that replicate using WT HIV reverse transcriptase or one of several mutants (L100I, K103N, V106A, E138K, Y181C, Y188L, H221Y and the double mutant K103N/Y181C) were used to infect cells in a single round assay. These mutants were chosen either because they are known NNRTI resistance mutations (L100I, K103N, Y181C, and the K103N/Y181C double mutant), they were selected in patients in rilpivirine clinical trials (E138K and H221Y), or they are positioned to make significant Van der Waals contacts with rilpivirine (V106A and Y188L). All compounds were initially screened against a panel that included at least one RT mutant from all three groups (WT, K103N, E138K, Y181C and Y188L). The cytotoxicity of the compounds was also determined to establish the therapeutic index of each of the compounds.

**Figure 3:**
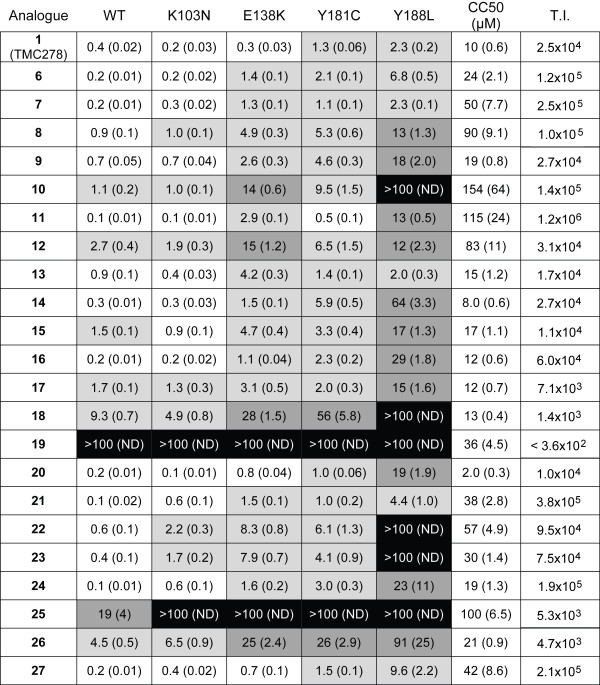
**Activities and cytotoxicities of rilpivirine (1) and its analogues.** EC_50s_, as measured by reduction of luciferase reporter activity, are reported as nM values, CC_50s_ are reported as μM values. Standard deviations are indicated in parentheses. Shading of EC_50_ values indicates a log scale: no shading = EC_50_ <1.0 nM, light gray = EC_50_ between 1.0 nM and 9.9 nM, dark gray = EC_50_ between 10 nM and 99nM, black = EC_50_ ≥100 nM. Therapeutic Index (T.I.) is the ratio of CC_50_/EC_50_.

Results from these analyses are shown in Figure 3. Rilpivirine (**1**) demonstrated potent activity versus a vector that replicates using WT RT (0.4 ± 0.02 nM) and each of the single RT mutants (the highest value was 2.3 ± 0.2 nM versus the Y188L mutant). The CC_50_ for **1** was 10 ± 0.6 μM. Pyrimidine analogues that retained the substituted aryl amines in the same structural location and had either a nitro or amine substitution added to the 5-position (Figure 2, analogues **6** and **7**) maintained similar activity profiles as **1** with only slight drops in activity versus the Y188L mutant (6.8 ± 0.5 nM and 2.3 ± 0.1 nM, respectively). A ‘flipping’ of this structural pattern (Figure 2, analogues **8** and **9**) resulted in a modest drop in potency against the WT RT and K103N mutant and showed more substantial loss of potency against E138K, Y181C and Y188L mutants.

### Inhibitory activity of purine-based analogues

The closest structural homolog to rilpivirine (**1**) among the purine derivatives (Figure 2, analogue **11**) retained good potency versus the WT as well as the K103N and Y181C mutants but showed a modest drop in activity versus the E138K mutant and a significant drop in activity versus the Y188L mutant (13 ± 0.5 nM). The ‘flipped’ analogue (Figure 2, analogue **13**) maintained potency similar to **1** for the WT and each of mutants with the exception of E138K (4.2 ± 0.3 nM). Retaining the p-methoxybenzyl protecting group at the 9-position of the purine reduced the potency of both analogues (Figures 2 and 3, compare **10** to **11** and **12** to **13**). Based upon these data, we used **13** as a scaffold and examined the effects of different substituents in place of the 4-benzonitrile moiety.

The results obtained with these analogues (Figure 2, analogues **14**–**25**) show that many of the compounds that are highly active against WT RT show considerable differences in their ability to inhibit the various mutants. Attempts to replace the nitrile functionality of the benzonitrile with other moieties resulted in compounds that were active against the WT and most mutants. However, these analogues (**14**–**19**) showed a significant loss of activity versus the Y188L mutant (Figure 3). Replacing the benzonitrile with alternate ring systems was generally successful against WT, but Y188L again conferred resistance (analogues **20**–**25**). The benzo[*d*]thiazole analogue (**21**) possessed particularly good activity versus WT and the mutants, including an EC_50_ of 4.4 nM against Y188L. The derivatives incorporating 2,9-purine ring systems (analogues **26** and **27**) were found to possess very different activities. Placement of the benzonitrile moiety on the 9-position resulted in an analogue (**26**) which showed a significant decrease in activity versus WT and all of the mutants. However, the incorporation of the (*E*)-3-(3,5-dimethylphenyl)acrylonitrile moiety at the 9-position resulted in a highly active analogue (**27**) with potencies akin to rilpivirine for WT and all of the mutants except Y188L, which caused a modest drop in activity (9.6 ± 2.2 nM).

### Selected analogues assayed against additional mutants

**Figure 4:**
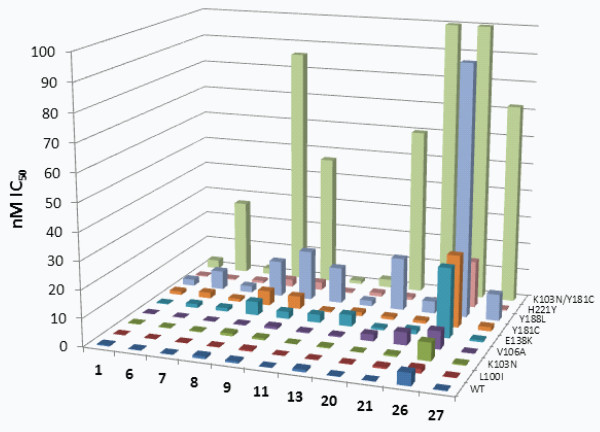
**Comparison of EC_50_ values of selected analogs against an expanded set of mutants.** Values for mutants not shown in Figure 3 are included in Additional file 1: Table S1.

Based on the results obtained with the small panel of mutants, eleven compounds were chosen for testing against a broader panel which included all the mutants listed above (K103N, E138K, Y181C and Y188L), and included rilpivirine (**1**) as a reference compound. The analogues **6**–**9**, **11**, **13**, **20**, **21**, **26** and **27** were selected based on their broad potency against the smaller panel of mutants and/or to determine how their differences from **1** affected their activities. The K103N/Y181C double mutant caused a significant loss of potency for **6**, **8**, **9**, **20**, **21**, **26** and **27** (Figure 4 and Additional file 1: Table S1), while the analogues **7**, **11** and **13** retained good potency against this mutant. Of particular interest, the related analogues **7** and **9** differed dramatically in their ability to inhibit K103N/Y181C RT (EC_50_ values of 2.2 nM and 47 nM, respectively). These compounds both possess a central 5-aminopyrimidine moiety that is ‘flipped’ between the two analogues. Keeping the pyrimidine ring in the same conformation as in **1** allows **7** to retain activity while **9** suffers ~67-fold loss of potency against the double mutant. A structural explanation for this difference will be discussed in a later section.

### Cytotoxicity

In general, the eleven analogues tested against the expanded mutant panel showed lower cytotoxicity compared to the other analogues (Figure 3). Only **20** had a lower CC_50_ than **1** (2.0 μM for **20** versus 10 μM for **1**). CC_50_ values for analogues **6**, **9**, **13** and **26** were 1- to 3-fold greater than that of **1**. The remaining compounds (**7**, **8**, **11**, **21** and **27**) had CC_50_ values greater than 38 μM (up to 115 μM for **11**). The resulting therapeutic indices (T.I., ratio of CC_50_ to EC_50_) ranged from 4.7x10^3^ (**26**) to 1.2x10^6^ (**11**). By comparison, rilpivirine (**1**) has a T.I. of 2.5x10^4^; so, based on the data obtained in cultured cells, many of these analogues compare favourably to this FDA-approved NNRTI.

### Crystal structure of 16 bound to WT RT

The crystal structure of the RT/**1** complex has been reported [11]. This structure shows that the (*E*)-3-(3,5-dimethylphenyl)acrylonitrile moiety occupied an induced hydrophobic tunnel formed between residues Y188, F227, W229 and L234. This moiety is retained in all analogues presented here. To examine the effects of modifications to other regions of the compound, a crystal structure of **16** bound to WT RT was solved at 2.3 Å resolution (Table 1).

**Table 1:** X-ray data and refinement statistics

	**HIV-1 WT RT/16**	**K103N/Y181C mutant HIV-1 RT/21**
Protein Data Bank (PDB) accession code	4I2P	4I2Q
Space group	C2	C2
Cell constants (a, b, c in Å; β in º)	162.65, 72.99, 109.50; 100.59	162.60, 72.95, 108.54; 100.70
Resolution range (Å)	50.00 – 2.30	50.00 – 2.70
Completeness (%)	98.8	99.2
R_merge_	0.085	0.084
Average I/σI	15.0	16.5
Sigma cut-off (I)	|I| < −3.0σ	|I| < −3.0σ
***Refinement Statistics***		
Total no. of atoms (solvent atoms)	8,258 (309)	8,094 (146)
Resolution (Å)	2.30	2.70
No. of reflections	55,924	34,219
R_work_	0.212	0.215
R_free_	0.260	0.286
Ramachandran statistics (% of residues in favored/disallowed regions)	99.48/0.52	99.90/0.10
RMSD bond length (Å)	0.009	0.009
RMSD bond angles (º)	1.120	1.258

**Figure 5:**
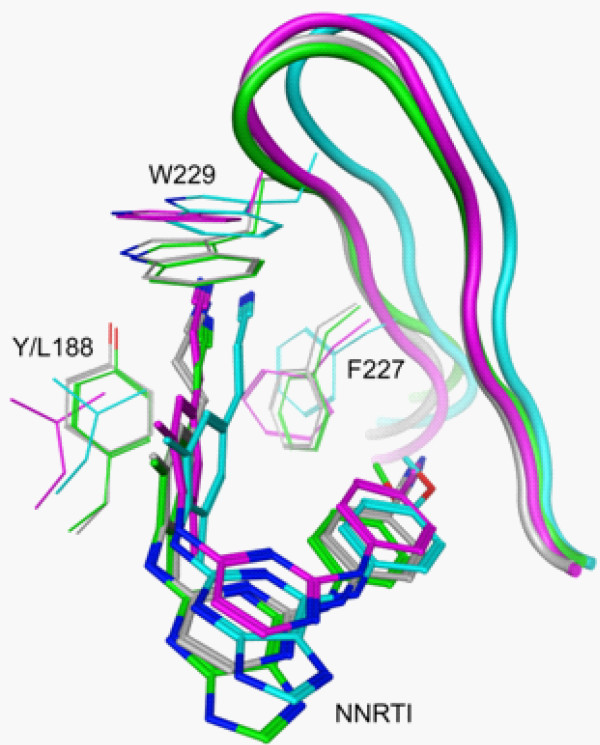
**Overlay of crystal structures and models of 1 and 16 bound to RT.** Co-crystal structures of WT RT bound to **1** (gray) and **16** (green) show only minimal differences in the position of the compound and the conformation of the binding pocket. This is consistent with the similar activity of the two compounds against WT RT (0.2nM for **1** and 0.4nM for **16**). Models were generated for each compound bound to Y188L RT (**1**: magenta, **16**:cyan). Both models show the compound positioned approximately one angstrom further into the binding pocket than in the respective WT co-crystal structure. This results in a repositioning of the F227 and W229 side chains and a shift in the overall positioning of the β12-β13 hairpin (shown as a ribbon). These differences in the modeled interactions are consistent with the observed difference in antiviral activities against vectors using Y188L RT (2.3nM for **1** and 29nM for **16**).

Overall, the two compounds bind WT RT very similarly (Figure 5). The (*E*)-3-(3,5-dimethylphenyl)acrylonitrile moiety occupies the hydrophobic tunnel as seen with **1**. In addition, the H-bonds between the central group (pyrimidine in **1**, purine in **16**) and the backbone amide and carbonyl groups of K101 are maintained. The ring of the 4-anisole group of **16** overlays with the ring of the 4-benzonitrile group of **1**. The most pronounced difference is between the bent nature of the methoxy functionality of 4-anisole (**16**) and the linear nature of the cyano functionality of 4-benzonitrile (**1**). This difference appears to have only a modest impact on the activities of the two compounds against WT RT (Figure 3).

Starting with the crystal structures of these two compounds bound to WT RT, models were generated of the complexes with Y188L RT. In both models, the compounds are shifted approximately one angstrom relative to their positions in the respective WT RT crystal structures. The linear nature of the cyano functionality of the 4-benzonitrile moiety in **1** allows it to extend past the side chain of F227 with only about 30º rotation around the Cβ-Cγ bond. The 4-anisole’s methoxy group (**16**) would cause a greater steric clash with the F227 side chain as it is positioned in the WT RT than would the 4-benzonitrile (**1**). As a result, a shift of the backbone of the β12-β13 hairpin is observed in the Y188L/**16** model relative to its position in the WT/**16** crystal structure (Figure 5). The F227 Cα is displaced by 1.2 Å and both the Cβ and Cγ atoms are displaced by 1.3 Å. This displacement is propagated throughout the loop including W229; the Cα atom of this residue is displaced by 2.1 Å. The repositioning of this hairpin provides a possible explanation for the observed loss of potency for **16** against Y188L RT.

### Crystal structure of 21 bound to K103N/Y181C RT

A structural study comparing rilpivirine binding to WT and mutant RTs revealed that the drug is able to retain its inhibitory activity by adapting its binding mode (wiggling and jiggling) to accommodate changes in the landscape of the binding pocket caused by NNRTI resistance mutations [11]. This hypothesis was tested by analyzing the structures of several mutant RTs with rilpivirine (**1**) bound and by comparing these binding modes to the binding modes of other NNRTIs, such as tert-butyldimethylsilyl-spiroaminooxathioledioxide (TSAO) [22]. To better understand why one of the rilpivirine analogues was not highly effective at inhibiting mutant RTs, we determined the crystal structure of the K103N/Y181C double-mutant RT in complex with **21** at 2.7 Å resolution (Table 1).

**Figure 6:**
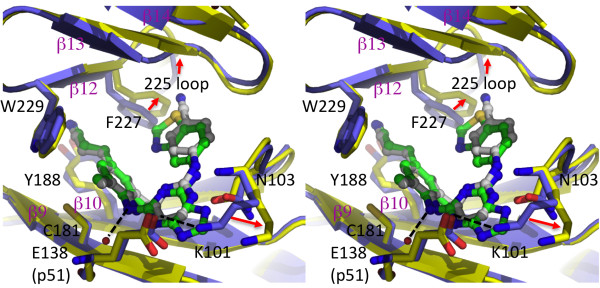
**NNRTI-binding pocket superposition of the crystal structures of K103N-Y181C mutant RT/21 (yellow protein and green ligand) and K103N/Y181C mutant RT/rilpivirine (PDB ID. 3BGR; blue protein and gray ligand).** The hydrogen bonds are represented as dotted lines, and the most significant structural differences are indicated by red arrows.

The double mutant K103N/Y181C RT confers ~8-fold resistance to riplivirine (**1**) and >1000-fold resistance to **21**. The overall binding mode of **21** (Figure 6) to the mutant RT is similar to and superimposes well with that of rilpivirine (**1**) [11]. The (*E*)-3-(3,5-dimethylphenyl)acrylonitrile moiety of **21**, common to both agents, occupies the hydrophobic core of the pocket and interacts with the aromatic side chains of Y188 and W229. Analogue **21** has a bulkier benzothiazole moiety substituted for the benzonitrile group of rilpivirine (**1**). As a result, the benzothiazole of **21** extends nearly 2 Å towards F227 and moved away from P236 compared to the nitrile group of **1**. The benzothiazole group of **21** has extended interactions with the side chains of V106, F227, and Y318. The side chain of F227 is reoriented to accommodate the benzothiazole group of **21**, has moved towards P225, and undergoes a ~40º rotation about the Cβ-Cγ bond relative to the RT-rilpivirine structure. Consequently, the flexible 222–226 loop is repositioned; however, the cis-peptide link between P225-P226 observed in the rilpivirine co-crystal is also present in the **21** co-crystal. The benzothiazole moiety of **21** does not have a significant impact on positioning of the β12-β13-β14 sheet, unlike that seen when TSAO or delavirdine binds to RT [22,23].

In **21**, the central pyrimidine ring of rilpivirine (**1**) is substituted by a purine that extends toward the side chains of K101 (p66) and E138 (p51) at the putative entrance to the inhibitor binding pocket. Both of these side-chains are involved with the entry/exit of NNRTIs into/out of the pocket, and a hydrogen bond exists between the two in most RT/NNRTI structures. In the double mutant K101E/E138K, the H-bond is retained, but these mutations cause a significant loss of potency to several preclinical TSAO-derived NNRTIs [22]. This suggests that the presence of specific amino acids at positions 101 and 138 may affect the binding of other NNRTIs. The positioning of the purine moiety of **21** causes significant rearrangements of the K101 and E138 side chains, which breaks the H-bond between these two residues and forms extensive hydrophobic contacts with methylene groups of both side chains. Interestingly, the N7 atom of the purine ring forms an H-bond with the backbone amide of K101 analogous to that seen with the pyrimidine ring of rilpivirine. The purine moiety also has extensive interactions with L100, the mutated K103N and V106. The mutated K103N side chain has more hydrophobic stacking interactions with the purine moiety of **21** than with the pyrimidine of rilpivirine (**1**). Our structure indicates that the benzothiazole moiety maintains inhibitor-protein interactions without requiring significant rearrangement of the pocket, apart from reorienting the side chains of K101, E138 (p51), and F227 (Figure 6). Also, the disruption of the K101-E138 H-bond may affect the dissociation of **21** in a manner that is not observable in the co-crystal. The positional constraints on the amino acid residues that are parts of the hydrophobic tunnel and the entrance to the pocket may explain why **21** is less susceptible to pocket mutations.

### Modeling other RTs and compounds

Several of the analogues showed significantly different activities against a particular RT mutant despite having only relatively modest structural changes relative to the parent compound. For example, compounds **7** and **9** differ only in the orientation of the central 5-aminopyrimidine moiety. This subtle change results in an EC_50_ for **9** that is ~21-fold greater than that of **7** against the K103N/Y181C mutant. Models of both of these complexes were generated from the crystal structure of **1** bound to this mutant [11].

**Figure 7:**
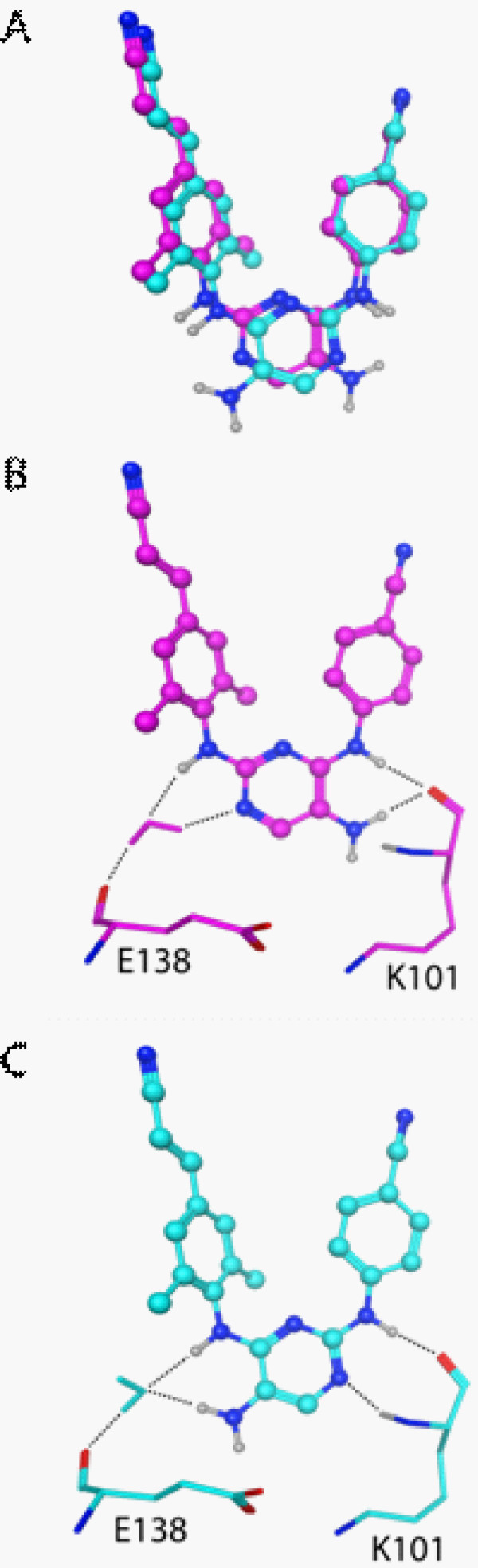
**Models of analogues 7 (cyan) and 9 (magenta) bound to K103N/Y181C RT.** (**A**) Overlay of the two compounds as modeled in the binding pocket of K103N/Y181C HIV-1 RT. (**B**) The exocyclic amine in **9** cannot H-bond with the backbone NH of K101, as seen in crystal structures of **1**. The N3 and linker NH both bind a water molecule that forms a bridge to the backbone carbonyl of E138. (**C**) The H-bonds with the backbone of K101 are restored in **7**. In addition, the exocyclic amine and linker NH both H-bond a water molecule in a different orientation than is seen with **9**.

The models show no significant changes to either the β12-β13-β14 sheet or the K101-E138 salt bridge. However, the solvent-exposed surface of the pyrimidine is shifted nearly an angstrom when the two models are compared to each other. This positions the exocyclic amine of **9** such that it cannot form the H-bond to the K101 backbone amide seen with **1** (Figure 7). In contrast, this H-bonding is maintained with **7**. These models also differ in the orientation of a water molecule forming a bridge to the backbone carbonyl of E138. The exocyclic amine and aryl amine of **7** serve as donors to bind the same oxygen atom of a water molecule. These interactions position the water differently than it appears in the model with **9**, where the pyrimidine’s N3 serves as an acceptor and the aryl amine as a donor to bind the same water molecule. The orientation of that water molecule could result in a weaker interaction with the E138 backbone. This subtle change in the compound, and the corresponding loss of activity against the K103N/Y181C mutant, highlights two potential interactions that need to be considered in the design of additional derivatives.

Intramolecular contacts also need to be considered. Analogues **6** and **8** have 5-nitropyrimidine central moieties in the ‘flipped’ orientations. These nitro groups appear capable of forming intramolecular H-bonds with the aryl amines bound to the 6-position of the pyrimidine ring. These interactions may restrict the flexibility of these analogues. This observation is consistent with the similar profiles of **8** and **26** and those of **6** and **27**. The potential intramolecular H-bonds may mimic the covalent restriction imposed by the purine ring systems of **26** and **27**.

## Conclusions

In summary, we have designed and synthesized several small molecules that are structurally related to the recently approved NNRTI rilpivirine (**1**) and tested them for their ability to inhibit the replication of viral vectors that carry WT RT or several clinically relevant mutant RTs. This data set provides insights into structure-activity relationships of this family of compounds. One agent, a purine analogue with a benzo[*d*]thiazole appendage at the 6-position (analogue **21**), possessed good activity versus all single mutants tested, but showed little activity against the K103N/Y181C double mutant. To understand the molecular basis of this loss of potency, we determined the crystal structure of the double-mutant K103N/Y181C RT in complex with **21**. This structure provided insights into how this analogue failed to maintain potency versus specific RT mutants. Molecular modeling also proved useful in assessing how other analogues interact with RT.

Three analogues were identified (**7**, **11** and **13**) that show nearly as broad potency and cytotoxicity comparable to, or more favourable than, that of rilpivirine (**1**). These three compounds showed some loss of potency (four- to fourteen-fold) against E138K and **11** lost some potency against Y188L (nearly six-fold). It is noted that rilpivirine is comparably active against vectors harbouring either WT or E138K RT in this assay despite the frequent selection of this mutant in clinical trials [13,24]. These trials used treatment regimens that included two NRTIs (TDF/FTC in the ECHO trial and TDF/FTC, zidovudine/lamivudine or abacavir/lamivudine in the THRIVE trial). E138K has been shown to compensate for the loss of viral fitness associated with certain NRTI resistance mutations, particularly M184I or M184V [15]. It is possible that the frequent selection of E138K is driven more by a need to restore fitness to NRTI-resistant RTs than by a significant affect on rilpivirine resistance, consistent with a higher rate of NRTI-resistant mutants selected in the rilpivirine group than in the efavirenz group in the clinical trials [13]. Controlling HIV infection must be done with combinations of drugs that work cooperatively against known resistant mutants. If any of these three analogues select for a different set of mutants than does rilpivirine, they may be useful in patients experiencing virologic failure on a rilpivirine regimen. Such selection experiments, and the synthesis and testing of additional analogues, represent future steps in this continuing effort.

## Methods

### Synthesis

All compounds shown in Figure 2 were synthesized and assayed for HIV inhibitory activity and cytotoxicity. Synthetic protocols and representative ^1^H NMR chemical shift data are included as Additional file 1. Briefly, aryl amines were coupled to a PMB-protected 2,6 dichloro-9H-purine starting reagent at the 6-position, followed by addition at the 2-position using a modified Buchwald-Hartwig amination to yield analogues **10-25**[25,26]. Analogues **26** and **27** were synthesized by modifying a synthetic protocol described in the patent literature [27].

### Virion production

Procedures for virion production and cell-based assay have been reported [28]. Briefly, all cell cultures were maintained in Dulbecco’s modified Eagle’s medium (Invitrogen, Carlsbad, CA) with 5% (v/v) fetal bovine serum, 5% newborn calf serum, penicillin (50 units/mL) and streptomycin (50 μg/mL) (Quality Biological, Gaithersburg, MD). VSV-g pseudotyped virions were produced by plating 9x10^5^ 293 T cells in a 100 mm plate, then transfecting with the pNLNgoMIVR^+^ΔEnv.LUC (10 μg) and pHCMV-g (3 μg) plasmids by calcium phosphate precipitation on the following day. Transfected cells were incubated at 37°C for 8 hrs, washed twice with PBS, fresh media was added and the cells were returned to the incubator for 48 hrs. The pNLNgoMIVR^+^ΔEnv.LUC plasmid encodes the HIV-1 genome with an Env deletion and either WT HIV-1 RT, or one of the following mutants: L100I, K103N, V106A, E138K, Y181C, Y188L, H221Y, or K103N/Y181C. Supernatants from each plate were clarified by low-speed centrifugation (3,000xg for 20 min), then treated with 200 U DNase I (Roche Applied Science, Mannheim, Germany) at room temperature for 3 hours. Viral supernatants were stored at −20°C until used.

### Infectivity assays

One day prior to infection, 100 μL of human osteosarcoma (HOS) cells were added to each well of a 96-well plate at a density of 4x10^4^ cells/mL. Plates were incubated overnight at 37°C. On the day of infection, 10X drug stocks were prepared by serial dilution in media from an initial 10 mM stock solution in DMSO. Cells in the 96-well plate were pre-treated by the addition of 22 μL of the appropriate drug stock and returned to the incubator for 3 hours. Thawed virus stocks were diluted 1:2 in media, then 100 μL of virus was added to the pre-treated cells, bringing the final drug concentrations to 0.1 nM to 100 nM, with DMSO-treated cells (no drug) serving as a reference. In addition, 12 wells of cells treated with media and no virus served as negative controls. Plates were returned to the incubator for an additional 42 hours, at which time the luciferase activity was determined using Steady-Lite Plus kits (PerkinElmer, Waltham, MA). Background luciferase activity, as measured for the negative controls, was subtracted from the activities measured for each infected well. Drug-free, DMSO-treated wells were normalized to 100% infectivity. Infectivity at each drug concentration was determined as a percentage of these drug-free infections. Drug concentration versus percent infectivity was plotted for each RT-inhibitor combination, and the data were fit to a four-parameter sigmoidal curve using KaleidaGraph 4.0 (Synergy Software, Reading, PA). The inflection point of each curve is reported as the IC_50_. All values are the mean of triplicate infections.

### Cytotoxicity assays

One day prior to treatment, HOS cells were prepared in 96-well plates as described for the infectivity assays, except that 12 wells per plate were treated with cell-free media to serve as negative controls. The following day, serial dilutions of drug stocks were prepared in media from 10 mM stocks in DMSO. Each well received 11 μL of the appropriate drug concentration or DMSO-treated, drug-free media. Final drug concentrations ranged from 0.5 μM to 300 μM. The drug-free wells served as references. Plates were then incubated at 37°C for 48 hours. Cell viability was determined using luciferase reporter ATP-Lite kits (PerkinElmer). As described for the infectivity assays, drug-free reference wells were normalized to 100%, and viabilities at different drug concentrations were determined as a percentage of these references. Data were plotted and analyzed in the same manner as the IC_50_ data, with the inflection points of the curves reported as CC_50_.

### Crystallography

RT was expressed and purified as described previously [29]. RT (WT and mutant) was co-crystallized with **16** and **21** at 4°C by vapor diffusion in micro-seeded hanging drops containing 1.2 μL each of 20 mg/mL protein (in a solution of 10 mM Tris pH 8.0, 75 mM NaCl, 0.30% (w/v) β-octyl glucopyranoside, 2% (v/v) DMSO, and 0.5 mM **16** or **21** pre-incubated for 10 min at 25°C) and a reservoir solution containing 50 mM imidazole pH 6.6, 100 mM ammonium sulfate, 15 mM manganese sulfate, 10 mM spermine, 5 mM TCEP, 11% (w/w) PEG 8000, and 5% PEG 400. The chosen crystals were soaked for ~20 s in a solution containing 50 mM imidazole pH 6.6, 50 mM ammonium sulfate, 15 mM manganese sulfate, 10 mM spermine, 12% (w/w) PEG 8000, 6% (w/w) PEG 400, and 26% (v/v) ethylene glycol. The crystal was subsequently flash-cooled and stored in liquid N_2_. Data collection was performed at the Cornell High Energy Synchrotron Source (CHESS) F1 beamline. The diffraction data were indexed, processed, scaled and merged using *HKL2000I*[30]*.* The structure was refined using Phenix [31] and model building was done using COOT [32]. The crystallographic data and refinement statistics are listed in Table 1.

### Computer modeling

All computer modeling was done using MOE 2009.10 or MOE2011.10 (Chemical Computing Group, Montreal, Quebec, Canada). The model of **1** bound to Y188L RT was based on the previously reported crystal structure of the WT RT/**1** complex (PDB ID: 2ZD1 [11]). Models of complexes with **16** and **21** were based on the crystal structures presented in the current work (PDB IDs 4I2P and 4I2Q, respectively). Models of **7** and **9** bound to K103N/Y181C RT were based on the K103N/Y181C RT/**1** crystal structure (PDB ID: 3BGR [11]).

## Competing interests

The authors declare that they have no competing interests.

## Authors’ contributions

BJ performed the infectivity and cytotoxicity assays and carried out the molecular modeling. GP, GR, HB, JS, DM and CT contributed to the synthesis of compounds. DP, JB, KD and EA performed the crystallographic studies. EA, CT and SH designed the experiments. BJ, KD, DM, EA, CT and SH drafted the manuscript. All authors read and approved the final manuscript.

## Supplementary Material

Additional file 1 **Synthetic schemes used to synthesize purine analogues of rilpivirine.** Scheme 1 was used to synthesize analogues **10**-**25** while Scheme 2 was used to synthesize analogues **26** and **27**. Click here for file
