# Supplementary material for: A comparison of the ability of rilpivirine (TMC278) and selected analogues to inhibit clinically relevant HIV-1 reverse transcriptase mutants
Source: Retrovirology. 2012 Dec 5;9:99. doi: 10.1186/1742-4690-9-99 (PMC3549755; doi:10.1186/1742-4690-9-99)
Supplement: Additional file 1 — Synthetic schemes used to synthesize purine analogues of rilpivirine. Scheme 1 was used to synthesize analogues 10-25 while Scheme 2 was used to synthesize analogues 26 and 27. [file 1742-4690-9-99-S1.doc]

**Additional Files**

**Synthesis**

The synthesis of purine-based analogues of rilpivirine (**1**) required a known PMB protected 2,6-dichloro-9H-purine starting reagent (Figure S1, Scheme 1).[30] Treatment with the specified aryl amine in *n*-butanol at 130 °C in a microwave reaction vessel afforded substitution at the 6-position of the purine ring in acceptable yields (61-78%). Displacement at the 2-position was accomplished using modified Buchwald-Hartwig amination reaction utilizing palladium (II) acetate, cesium carbonate and 2,2′-bis(diphenylphosphino)-1,1′-binapthyl *(*BINAP) in toluene heated to 110 °C in a sealed reaction vessel for a 10 h period to provide the di-substituted, PMB protected precursors (**10**, **12**) in acceptable yields (39-57%).[31] Deprotection of the PMB group was accomplished via treatment with a TFA/CH_2_Cl_2_ solution and microwave irradiation to 100 °C for a period of 30 minutes to provide a range of analogues (**11**, **13**-**25**).

We also examined the key aromatic moieties on the 9 position of the purine ring system to compare with the effects of these substitutions with the standard *N^2^*,*N^6^*-diaryl substituted analogues. The synthetic sequence to prepare these analogues was adapted from the patent literature.[32] It was initiated by reacting commercially available 2,4-dichloro-5-nitropyrimidine with the requisite aryl amine utilizing a neat reaction at 140 °C (Figure 7, Scheme 2). The 2-chloro position was subsequently substituted with the second aryl amine moiety via microwave heating at 110 °C in DMF. The aromatic nitro group was reduced utilizing stannous chloride in ethanol at elevated temperature to produce the aniline derivative in good yield. The purine ring was formed via treatment with triethylorthoformate (neat) at 100 °C to provide the desired agents for biochemical analysis (analogues **26** and **27**). All of the analogues were purified via preparative scale reverse-phase HPLC. Each analogue was confirmed for purity via separate LCMS runs utilizing different gradients, proton NMR and HRMS analysis. Representative proton NMR data for selected compounds are shown below.

**7:** (E)-4-((5-amino-4-((4-(2-cyanovinyl)-2,6-dimethylphenyl)amino)pyrimidin-2-yl)amino)benzonitrile. ^1^H NMR (400 MHz, DMSO-*d*_6_) 2.17 (s, 6 H), 6.54 (d, J=16.7 Hz, 1 H), 7.38-7.31 (m, 4 H), 7.57 (s, 3 H), 7.71 (d, J=16.4, 1 H), 9.51 (bs, 1 H) and 10.42 (bs, 1 H); HRMS (ESI) *m/z* (M+H)^+^ calcd. For C_22_H_19_N_7_, 382.1775; found 382.1769.

**13:** (E)-4-((2-((4-(2-cyanovinyl)-2,6-dimethylphenyl)amino)-9H-purin-6-yl)amino)benzonitrile. ^1^H NMR (400 MHz, DMSO-*d*_6_) 2.20 (s, 6 H), 6.46 (d J=16.4 Hz, 1 H), 7.48 (s, 2 H), 7.51 (bs, 2 H), 7.66 (d J=16.4 Hz, 1 H), 7.97 (bs 2 H), 8.23 (s, 1 H), 8.78 (bs, 1 H) and 10.29 (bs, 1 H); HRMS (ESI) *m/z* (M+H)^+^ calcd. For C_23_H_18_N_8_, 407.1727; found 407.1721.

**16:** (E)-3-(4-((6-((4-methoxyphenyl)amino)-9H-purin-2-yl)amino)-3,5-dimethylphenyl)acrylonitrile. ^1^H NMR (400 MHz, DMSO-*d*_6_) 2.17 (s, 6 H), 3.71 (s, 3 H), 6.50 (d, J=16.8 Hz, 1 H), 6.67 (bs, 2 H), 7.47 (bs, 2 H), 7.67 (d, J=16.8 Hz, 1H) 8.4 (s, 1 H), 9.21 (bs, 1 H) and 10.10 (bs, 1 H).

**21:** (E)-3-(4-(6-(benzo[d]thiazol-5-ylamino)-9H-purin-2-ylamino)-3,5-dimethylphenyl)acrylonitrile. ^1^H NMR (400 MHz, DMSO-*d*_6_) 2.21 (s, 6 H), 6.40 (d, J=16.6 Hz, 1 H), 7.44 (s, 2 H), 7.60 (d, J=16.6 Hz, 1 H), 7.90 (bs, 2 H), 8.14 (bs, 2 H), 8.58 (bs, 2 H), 9.32 (s, 1 H) and 9.89 (bs, 1 H); HRMS (ESI) *m/z* (M+H)^+^ calcd. For C_23_H_18_N_8_S, 439.1448; found 439.1445.

**27:** (E)-4-((9-(4-(2-cyanovinyl)-2,6-dimethylphenyl)-9H-purin-2-yl)amino)benzonitrile. ^1^H NMR (400 MHz, DMSO-*d*_6_) 2.02 (s, 6 H), 6.58 (d, J=16.8 Hz, 1 H), 6.64 (d, J=9.2 Hz, 2H), 7.69 (d, J=16.8 Hz, 1 H), 7.94 (d, J=9.2 Hz, 2 H), 8.48 (s, 1 H), 9.10 (s, 1 H) and 10.29 (s, 1 H).

**Figure S1.** Synthetic schemes used to synthesis purine analogues of rilpivirine.

| Analogue | WT | L100I | V106A | H221Y | K103N/Y181C |
| --- | --- | --- | --- | --- | --- |
| **1** | 0.4 (0.02) | < 0.1 (ND) | 0.1 (0.01) | 0.4 (0.04) | 3.1 (0.1) |
| **6** | 0.2 (0.1) | < 0.1 (ND) | 0.1 (0.01) | 0.4 (0.04) | 27 (2.2) |
| **7** | 0.2 (0.01) | 0.1 (0.01) | 0.1 (0.01) | 0.9 (0.08) | 2.2 (0.1) |
| **8** | 0.9 (0.1) | 0.2 (0.02) | 0.3 (0.04) | 2.6 (0.4) | 86 (14) |
| **9** | 0.7 (0.05) | 0.5 (0.04) | 0.9 (0.2) | 2.8 (0.3) | 47 (1.6) |
| **11** | 0.1 (0.01) | < 0.1 (ND) | 0.4 (0.03) | 0.4 (0.02) | 1.2 (0.04) |
| **13** | 0.9 (0.1) | 0.1 (0.01) | 0.5 (0.02) | 1.3 (0.1) | 2.9 (0.1) |
| **20** | 0.2 (0.01) | 0.2 (0.02) | 2.1 (0.1) | 1.1 (0.04) | 60 (1.7) |
| **21** | 0.1 (0.02) | 0.6 (0.06) | 4.6 (0.3) | 0.6 (0.04) | >100 (ND) |
| **26** | 4.5 (0.5) | 1.6 (0.3) | 6.5 (1.2) | 17 (1.6) | >100 (ND) |
| **27** | 0.2 (0.01) | 0.2 (0.02) | 0.2 (0.02) | 0.4 (0.01) | 72 (2.2) |

**Table S1.** EC_50_ values (nM) of analogues tested against broader panel of mutants. As in Table 1, EC_50_ values are reported as the inhibitor concentrations causing a 50% reduction in luciferase reporter activity. Shading is the same as in Figure 3. Standard deviations are shown in parentheses.
